# A Multimethodology Contractor Assessment Model for Facilitating Green Innovation: The View of Energy and Environmental Protection

**DOI:** 10.1155/2013/624340

**Published:** 2013-11-07

**Authors:** Sung-Lin Hsueh, Min-Ren Yan

**Affiliations:** ^1^Graduate Institute of Cultural and Creative Design, Tung Fang Design Institute, No. 110 Dongfang Road, Hunei District, Kaohsiung City 82941, Taiwan; ^2^Department of International Business Administration, Chinese Culture University, No. 231, Sec 2, Jian guo S. Road, Da-an District, Taipei City 106, Taiwan

## Abstract

The trends of the green supply chain are attributed to pressures from the environment and from customers. Green innovation is a practice for creating competitive advantage in sustainable development. To keep up with the changing business environment, the construction industry needs an appropriate assessment tool to examine the intrinsic and extrinsic effects regarding corporate competitive advantage. From the viewpoint of energy and environmental protection, this study combines four scientific methodologies to develop an assessment model for the green innovation of contractors. System dynamics can be used to estimate the future trends for the overall industrial structure and is useful in predicting competitive advantage in the industry. The analytic hierarchy process (AHP) and utility theory focus on the customer's attitude toward risk and are useful for comprehending changes in objective requirements in the environment. Fuzzy logic can simplify complicated intrinsic and extrinsic factors and express them with a number or ratio that is easy to understand. The proposed assessment model can be used as a reference to guide the government in examining the public constructions that qualified green contractors participate in. Additionally, the assessment model serves an indicator of relative competitiveness that can help the general contractor and subcontractor to evaluate themselves and further green innovations.

## 1. Introduction

To address the issue of high greenhouse gas emissions, numerous improvements and policies have been developed for the construction industry, such as incentive policies for green architecture, energy-saving building materials, and green procurement. Considering that the construction industry is a high-pollution industry that influences overall economic development significantly, incentives are provided to stimulate the adoption of new policies, and they can be regarded as part of the agenda to transform the entire construction industry supply chain to a green construction industry. Currently, contractors with green construction qualifications are required throughout all stages of public engineering projects, from design, contracting and procurement, and construction technology and management to use and maintenance stages. In the context of strengthening the green building policy, transformation is inevitable for traditional constructors. The value of this study is that it provides performance assessments for companies' efforts to implement green transformation and green innovation and alleviates the impacts to enterprises that result from rapid business environment changes. The construction industry is a business sector that generates serious environmental pollution [1–5] and consumes enormous amounts of energy [6, 7]. Based on an estimate by the United Nations Environment Program, the building sector accounts for 30 to 40% of global energy use [8]. As global warming (climate change) becomes more serious [9, 10], many companies have adopted a proactive strategy [11]. Public environmental policy inspired the way in which firms act to adapt to challenges related to the natural environment and the subsequent advancements in environmental performance of the firms [12]. These strategies, such as the management of greenhouse gas emissions in large European companies [13], and the integration of suppliers into the environmental management processes proposed by Walton et al. [14], have had positive effects on environmental protection. Thus, improving construction practices to minimize their detrimental effects on the natural environment has been an emerging issue [15, 16]. Additionally, the environmental impact of construction green building, design for recycling, and the ecolabeling of building materials have captured the attention of building professionals across the world [17–19].

Because the environment cannot bear the pressure of excess energy consumption, and due to greater emphasis on the issue of environmental protection and the concept of using green products, suppliers have faced increasing pressure from their customers to improve their environmental performance [20]. The pressure also urges all sectors around the world to focus on the development of green supply chain management (GSCM) [21–23]. The development of the green sector has become an effective measure in resuscitating the economy and increasing the employment rate [24]. The EU has been very proactive and practical in environmental protection and the economic development of the green sector. In the Green Book of the EU, corporate social responsibility is a major tool for creating new jobs and sustaining economic development [25]. Corporate social responsibility, which is important to environmental protection, plays an important role when a corporation pursues profit and facilitates economic development [26, 27]. Many corporations have recognized that the corporate social responsibility is also the source of competitive advantage for future business opportunities [28, 29]. Green innovation, therefore, is not only the social responsibility of corporations, but also a practice of creating competitive advantages in a sustainable business [2, 21].

Managers proactively develop their unique capabilities to gain advantage over their competitors [30] and sometimes sacrifice profits only to improve their relative competitive standing [31]. In developed countries, the trends in development of construction industries have been focusing on green innovation, with an emphasis on mitigating the impact on the environment, whether in constructing supply-chain management, design and implementation, materials and facilities, or procurement. On a foundation of sustainable development, the construction industry must emphasize social responsibility while taking responsibility for protecting the environment. Currently, the movement toward environmental management systems is gaining momentum in the construction industries of most developed countries. However, this field is still relatively new, and its concepts are still marginalized in most of the developing world [5]. This new concept has conveyed an important message; namely, that green innovation is the key to the business transformation and future survival of the traditional construction industry. If a traditional general contractor and its subcontractors are not familiar with green business, including green materials, green contracts, green implementation, and green management they are likely to lose market share and competitiveness over time. Mistakes due to unfamiliarity with green requirements in an ongoing construction project, such as incorrect estimations, procurements, implementation, and delays in schedule, could also lead to profit loss.

Taiwan promulgated the government procurement act in 1999. The law has provided foreign contractors with a fair opportunity to bid in the Taiwanese construction market. In 2008, the Taiwanese government further relaxed the restrictions for international bidding, which has brought stricter pressure from global competition in the Taiwanese construction industry. The Taiwanese construction industry must realize the importance of industrial upgrading and transformation [32] and be proactive and responsive to changing markets to increase overall international competitiveness [33]. Over the past ten years, the Taiwanese government has issued many new building acts, such as the sustainable construction [34], the construction automation plan [35], regulations for green buildings, and examples for improvement to encourage private green buildings [36]. These acts aimed at upgrading the techniques and international competitiveness for the Taiwanese construction industry, while promoting green development and environmental protection. Those contractors which only possess traditional construction capacities can benefit from learning new techniques and implementing green transformation under the environment of green development.

The Taiwanese government has issued numerous green policies to increase the global competitiveness of the construction industry. The policies not only require promotion and proper execution but also need a method for measuring policy effectiveness. However, many issues still exist in designing an appropriate model to assess contractor competitiveness due to green innovation, such as the future development and trends in the overall industrial structure, trends in environmental factors, changes in customer preferences, advances in techniques and technology, and the changes in policy. Therefore, multiple methodologies, including system dynamics, the AHP, utility theory, and fuzzy logic theory, were used to increase the preciseness of the model and ensure its reliability. As an example, the ecological environment in the Taiwanese construction industry was presented to illustrate the use of the model and to demonstrate its capability.

## 2. Model Overview

The method of system dynamics, combining the feedback system of cybernetics and the engineering theory in servo-mechanism, was developed by Dr. Jay W. Forrester of the Massachusetts Institute of Technology (MIT) in 1960. The term “feedback” represents the process by which a specific signal travels through the causal chain and finally feeds back to itself [37]. System dynamics can precisely analyze complicated problems for decision making and thus is widely used in various areas as follows: sustainable land use and urban development [38]; environmental sustainability [39]; the interaction between environmental and economic factors in the mining industry [40]; sustainability in electrical and electronic equipment closed-loop supply chains [41]; remanufacturing in closed-loop supply chains [42]; forecasting the natural gas demand in China [43]; and energy consumption in iron and steel industry [44]. Based on these research areas, it is indicated that system dynamics is a specialized method for solving fundamental industrial system problems and thus is used to facilitate the sustainable development of all sectors.

Fuzzy theory is a concept based on the set theory proposed by Zadeh [45–47]. Fuzzy logic is able to tolerate ambiguities in natural human language, such as uncertainty, complexity, and tolerance for imprecision [48, 49]. The fuzzy set theory can be used in a wide range of domains in which information is incomplete or imprecise; such as using natural language to indicate “good,” “bad,” “like,” and “dislike.” Additionally, membership functions provide a means of quantifying the meaning of linguistic values where the degree of membership of an element in a given set is denoted by having values between 0 and 1 [50]. Studies have investigated the applications of fuzzy logic theory to decision making in the construction industry and other fields, including evaluating new construction technology [51], selecting an architecture-engineering team [52], selecting the most efficient maintenance approach [53–55], the evaluation of industrial robotic systems [56], developing the bargaining decision support model [57], and analysis of energy consumption [58]. Fuzzy theory lends itself to areas of decision making that are difficult to quantify or complicated to assess [59], especially the problem of group decision making [60–63].

The analytical hierarchy process (AHP) method has been widely used for solving decision making problems with multiple criteria [64]. The AHP was first proposed by Saaty. It is widely used in the decision making on social, political, and engineering issues and selection of intelligent building systems [65–68]. 

Utility is a term used in economics. It originated with English scholar Jeremy Bentham, and it can measure the preferences of consumers and serve as a unit of personal welfare. The utility function can represent the preference and relative attitude toward risk of consumers [69]. Research that has applied the utility theory includes green supply chain management [70], joint ventures in construction [71], design-build projects [72], bid markup decisions [73], and evaluating BOT projects [74, 75]. The utility function has several advantages when used to construct an assessment model. It can not only overcome the difficulties of building a multicriteria model, but can also support decision makers to make proper adjustments according to their preferences and risk attitude, so as to reduce inconsistent decisions influenced by various factors, such as emotions, the environment, and information [71].

**Figure 1:**
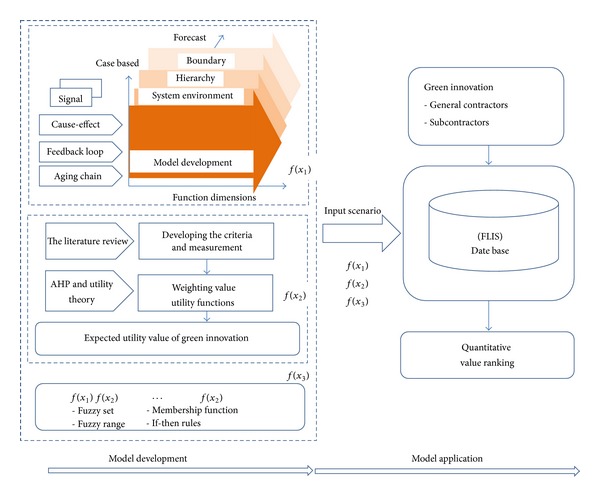
The proposed assessment model.

This study combined four methodologies—system dynamics, fuzzy logic theory, AHP, and utility theory—and developed a multimethod competitive advantage assessment model for the green innovation of contractors. As the issue of environmental protection becomes more important in the construction industry, corporations can use this model to evaluate and create competitive advantages. During the modeling process, the causal relationships in the overall industrial structure and the complicated factors in the system environment were considered; thus, the model is highly precise and reliable. Therefore, the proposed model is applicable under rapid changes in the market environment. The model of comprised two parts: (1) Model development, and (2) model application, as shown in Figure 1.

## 3. Model Development 

The assessment model consisted of two parts: (1) model development, and (2) model application. During model development we confirmed that there were three main input criteria to the fuzzy logic inference system (FLIS): *f*(*x*_1_), *f*(*x*_2_), and *f*(*x*_3_). Assessment of the content of these three criteria is described as follows: *f*(*x*_1_) was used to assess the future growth in the number of contractors in Taiwan, such that the overall competition in the Taiwanese construction industry can be evaluated; *f*(*x*_2_) was used to assess the effectiveness of green innovation in the Taiwanese construction industry; and *f*(*x*_3_) was used to assess the social responsibility of contractors, because it has been shown that social responsibility affects corporate competitiveness [28, 29]. Because there exists a large variation between the assessment content and the informational attributes associated with the three criteria, four methodologies were used to solve the problem.

### 3.1. Applying System Dynamics to Examine the Informational Attributes of Criterion *f*(*x*_1_)

System dynamics is mostly used to handle overall system structures that are difficult to explain. The modeling approach can take on a different form based on the properties of the system in question. The aging chain is specialized in the time-related simulation and control of inventory and flow in system dynamics, so it is suitable in examining future trends in the number of contractors in Taiwan.

According to the regulations for the management of contractors listed in Table 1, contractors are rated as class A, class B, or class C. The main factors affecting the rating for a contractor are revenue and capital. Therefore, the study focused only on these two factors.

**Figure 2:**
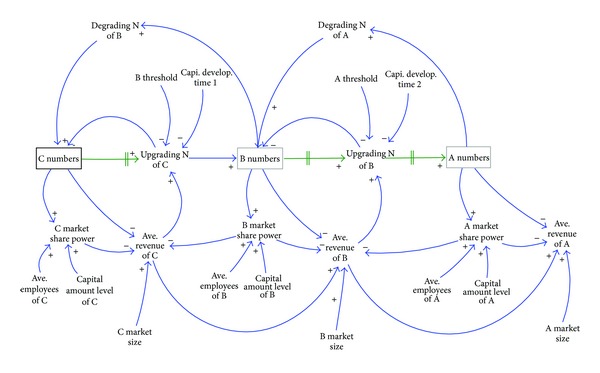
The casual loop diagram of the contractor rating system.

Based on the ratings in Table 1, the causal loop diagram (CLD) of the contractor rating system was shown in Figure 2.  Table 2 listed the meanings of all the variables used in Figure 2. Class A, B, and C contractors were simplified as A, B, and C, respectively. It was shown in Figure 2 that the contractor rating system could be represented by three inventory structures, C numbers, B numbers, and A numbers, which represent the number of contractors in class C, class B, and class A, respectively. The inventory structures have a relationship through the aging chain, in which contractors of a lower class can be rolled into the next higher class, as the time and revenue amounts reach the requirements for said class. The growth rate for the contractor is affected by its revenue required, capital, and average revenue. The higher its average revenue, the greater its growth. The annual revenue of a contractor has a direct relationship with the size of the construction market and with the number of contractors in competition. Theoretically, if market revenue is uniformly distributed to each contractor then the average revenue for each contractor should be equal to total market revenue divided by the total number of contractors. However, the construction market is a collection of competitors in which market revenue is not uniformly distributed. In fact, those contractors who are more competitive in the market or have a better capability for undertaking contracts are always awarded more contracts. To account for this fact in the model, in this study we assumed that the more capital and employees a contractor has, the better its capability to undertake contracts is. The rationales are as follows:

1. Contractors of a large scale in terms of capital or number of employees have competitive advantages, such as superior techniques, lower costs, or superior qualifications, which lead to more opportunities to undertake contracts.
2. Large scale contractors are responsible for more regular expenditures than medium or small scale contractors. This also makes the contractors of a large scale more proactive in undertaking more contracts to keep up with their expenditures.

Therefore, in this paper, we propose that the annual amount of revenue for a contractor is affected by its “market share power.” This term is defined as “the capability of a contractor to control the undertaking of contracts.” The more market share power the contractor is, the more contracts the contractor can undertake. Lastly, class degradation in the contractor rating system was shown in the upper half of Figure 2. The diagram shown in Figure 2 consisted of the reinforcing and balancing loops which are intertwined to form a causal relationship.

**Figure 3:**
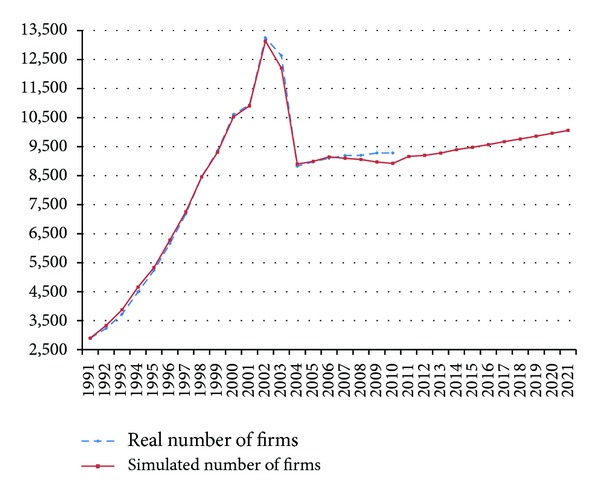
Comparison between simulated and actual numbers of contractors.

The data used to verify the model was based on actual information from the Council for Economic Planning and Development of Taiwan, Construction and Planning Agency of the Ministry of the Interior (CPAMI), and statistics from 1991 to 2009 of the Taiwan Construction Association [76–78]. This information showed the trend in the number of contractors in the Taiwanese construction industry. The system dynamics model was simulated from 1991 to 2009, as shown in Figure 3. In this figure, the *x*-axis denotes the year, and the *y*-axis denotes the number of contractors. The simulated result showed a close match with the actual number of contractors. The statistics of the past twenty years show that when the number of contractors reached 13,500, market competition was severe, and thus many contractors went out of business. The construction market is more business friendly to the contractors when there are fewer than 7,500 contractors, while indicators of competition start to increase when there are over 9,000 contractors. Based on the system dynamics model, future trends in the number of contractors in Taiwan can be predicted. If the construction market maintains normal conditions, the number of contractors will grow over time. The number of contractors has been limited to 9,000 over the past several years; however, in 10 years, it is most likely that the number of contractors will once again surpass 10,000. At that time, the construction market will become more competitive, and a direct impact on contractor profits is expected.

The statistics of the actual number of contractors in Table 3 show that following the peak of total contractor numbers in 2002, the construction industry in Taiwan displayed a trend of destructive competition and overgrowth, as well as significant risk of bankruptcy. As a result, large, listed construction companies were among the companies that declared bankruptcy at the time. Precisely determining development trends in the number of contractors in Taiwan during the past 20 years, the proposed system model in this study can be used as a reference for estimating the growth of contractor numbers as well as their inherent competitive strengths. 

Following the stringent requirements of the system dynamics methodology, the research model in this study is developed through careful and detailed processes from the definition of the research scope to the configuration of variables and parameters, causal relationships between variables and mathematical expressions, data collection, and model reliability testing and validation. Aside from the key points, among which, one of the most important topics is reliability testing and result validation, all details cannot be listed because of the limited scope of this paper. This study gathered real-world data on the changes in the number of construction contractors over the past 20 years in Taiwan and analyzed the results in comparison with the simulated results from the proposed model. Through statistical calculations of error mean square (*R* ^2^) and minimum absolute percentage error (MAPE), the results indicate that dynamically simulated estimates employing the proposed model can achieve excellent precision, and therefore the configuration of the model meets the requirements for research.

### 3.2. Applying the AHP and Utility Theory to Examine the Informational Attributes of Criterion *f*(*x*_2_)

Promoted by developed countries due to their emphasis on environmental issues, green development has become a fundamental requirement for sustainable development and creating competitive advantages in the construction industry. To this regard, the Taiwanese government has issued many policies, including green development, to increase international competitiveness in the construction industry. In terms of technology and capital scale, contractors in Taiwan do not have experience in undertaking large-scale international construction contracts. Compared to contractors in developed countries, the contractors in Taiwan lack in international competitiveness. Additionally, most of the mid- or small-scale contractors in Taiwan are still taking a traditional construction approach. It is likely difficult to implement an overall upgrade in technology and a green transformation in an industry within a short period. However, the Taiwanese government has issued many policies in helping the construction industry to improve and has relaxed regulations on international bidding in the law of procurements. These acts help the construction industry in Taiwan to acquire new construction knowledge and techniques and are beneficial to firms in upgrading or engaging in green development.

In the construction industry, contractors have been confronting the challenge of meeting the emerging needs related to the reduction of environmental impacts during the construction process [79]. Green development and green innovation have become the global trends in the construction industry. However, there exist many factors affecting green development and green innovation. Issues such as design, procurement, implementation, and management should be considered. The related influential factors are summarized as follows: multidesign [80], reduction in energy consumption [81], green walls and green roofs [82], green building [83], green open spaces [84–86], green procurement [87], green construction management [79], disposal of waste building materials [88], green specifications/green technology [8], and the green supply chain in the construction industry [89]. In addition, complying with government policies is also one of the influential factors. Based on these influential factors, 50 effective AHP questionnaires were collected to obtain the weighting value of each criterion.

**Figure 4:**
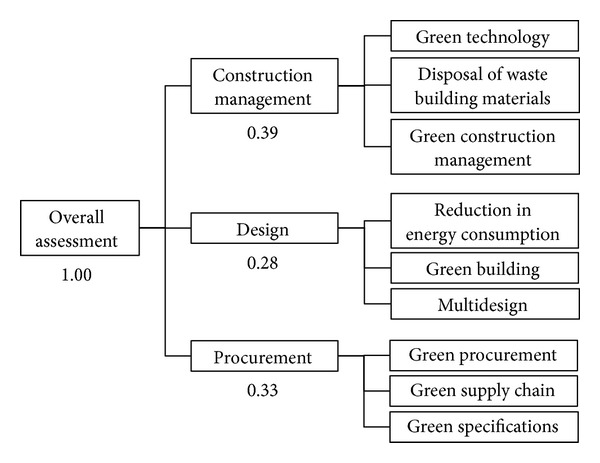
The AHP architecture of each criterion.

The AHP architecture of this study was developed using the Delphi Process (Figure 4). Although the calculation of the weights of the AHP questionnaire can be performed relatively quickly using Microsoft Excel, the AHP process is relatively long. Hsueh reported that it took over a year to complete the AHP process [71]. In addition, the AHP questionnaire was commissioned to professionals for assistance, and thus a complete and effective survey result can be expected. The AHP process in this study was completed using a stringent research attitude. In particular, 50 respondents who offered their assistance for AHP process research by completing a valid questionnaire were experts with over 15 years of work experience in relevant fields. Experts included famous vice-presidents, deans, experienced professors from the top five universities in Taiwan for the scholar portion, architects, CEOs, project managers with master's degrees for the industry portion, and government officials with master's degrees for the government portion. The AHP process required two years to finish. The weighting of each criterion are listed in Tables 4, 5, 6, 7, and 8.

The AHP is only capable of obtaining the relative weights between the factors, but if it is combined with utility, it can obtain the expected utility value: a quantitative value that can compare size and has reference value for decision making. Therefore, the expected utility value is an important parameter for the FLIS in this study.

The utility theory determines the function for each criterion and its risk value range, based on the experience and preferences of a decision maker. In this paper, a utility function with a linear relationship was used to create the utility function for each criterion [73]. Each criterion is associated with a utility linear function, *u*_*i*_(*y*_*i*_) = *Ay*_*i*_ + *B*; where the fuzzy scale for each criterion is within (*y*_*u*_, *y*_*L*_); *y*_*ma*_ is the point most preferred and *y*_*mi*_ is the worst point. Because *u*_*i*_(*y*_*mi*_) = 0; *u*_*i*_(*y*_*ma*_) = 1, we can obtain the following equations:

(1) $$\begin{array}{c} u_{i}\left(y_{mi}\right)=A\times y_{mi}+B=0,\;B=-Ay_{mi},\\ u_{i}\left(y_{ma}\right)=A\times y_{ma}+B=1,\;A=\frac{1}{\left(y_{ma}-y_{mi}\right)}. \end{array}$$

The expected utility value equals the sum of relative ratings *u*_*i*_(*y*_*i*_)* weighting value (*W*_*i*_) of each criterion and could be indicated by the following equation (*u*_*i*_(*y*_*i*_) = *u*_*ri*_):

(2) $$\begin{array}{c} \text{expected}\;\text{utility}\;\text{value}\;\left(\text{EUV}\right)=\overset{n}{\underset{i=1}{\sum }}\left(u_{ri}\times W_{i}\right). \end{array}$$

The AHP and utility theory were used to compute *f*(*x*_2_). The information attributes are shown in Table 9. The results suggest that the higher the EUV is, the more capable the green transformation is and the better the green innovation for a contractor is, such that the contractor can be more profitable even when the market changes.

### 3.3. Informational Attributes of Criterion *f*(*x*_3_) and the Fuzzy Logic Inference System

**Figure 5:**
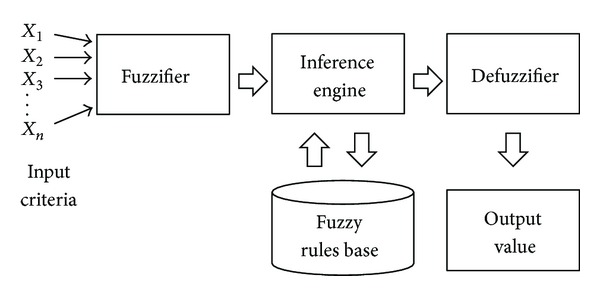
Diagram of the FLIS.

The core of the fuzzy logic model computation is FLIS, an artificial intelligence model that places all input combinations through the fuzzifier; the inferences of the rule base then pass through the defuzzifier; and the quantification transformation process is completed. The schematic diagram of FLIS is shown in Figure 5. In addition, for the establishment of the FLIS, the membership functions should be selected, and the fuzzy scale should be defined first [59, 90, 91]. In the fuzzy scale definition section, the fuzzy scale of the assessment factors for the number of constructors was used to determine the development trend of the number of constructors in Taiwan through systematic dynamics, which reflects inherent competitive power. The fuzzy scale of the assessment factors of green innovation calculates the range value of the expected utility by applying the AHP and utility theory and presents the quantified utility value of green innovation in an enterprise. Initially introduced by D. Bernoulli in 1738, the utility theory is a quantitative tool to analyze human values [92, 93]. With respect to applications for decision making and risk assessment, Hsueh et al. [71] successfully established a risk assessment model for construction joint ventures in China using the AHP and utility theory. Furthermore, the fuzzy scale of the assessment factors for corporate social responsibility concerns the adaptability of businesses to government policies and their perceptions of environmental protection. We define the scale value by using the fuzzy theory tolerance for imprecise semantics of natural human language.

Therefore, system dynamics, the AHP, and utility theory were used to compute the quantitative data attributes for the criteria *f*(*x*_1_) and *f*(*x*_2_). As for criterion *f*(*x*_3_), which corresponds to corporate social responsibility, fuzzy logic theory was applied to compute its quantitative informational attributes. Fuzzy logic theory is specialized in the uncertainty, complexity, and tolerance for imprecision used in natural language. The quantitative transformation of criterion *f*(*x*_3_) was based on the words “good,” “ordinary,” or “poor” used in natural language. The fuzzy logic model needs the if-then rules as a basis and the fuzzy logic inference system (FLIS) to possess the capability for inference. An effective fuzzy definition and precise informational attributes increase the reliability of the model. The informational attributes and the fuzzy definitions of the three criteria are listed in Table 10. The informational attributes for the three criteria, including the input and output fuzzy sets and fuzzy scale, were precisely defined. Moreover, commonly used membership functions include triangular functions and bell-shaped functions [94]. In this paper, triangular functions and bell-shaped functions were also used.

## 4. Model Application and Case Study

The main purpose of this section is to present the applicability of the research model. Therefore, the key is to present the examples of application with the research model, with details being provided as follows.

There are three input scenarios for criterion *f*(*x*_1_), five input scenarios for criterion *f*(*x*_2_), and three input scenarios for criterion *f*(*x*_3_). Therefore, the number of input scenarios for assessing a contractor's competitive advantage affected by green innovation is 45 (3∗5∗3). The data attributes of the three criteria are not different; however, the output of the quantitative ratio of profit was computed through defuzzification in the FLIS. On the ground of environmental protection, the ratio of profit can provide the contractor with a self-assessment tool in green transformation and green innovation.

**Figure 6:**
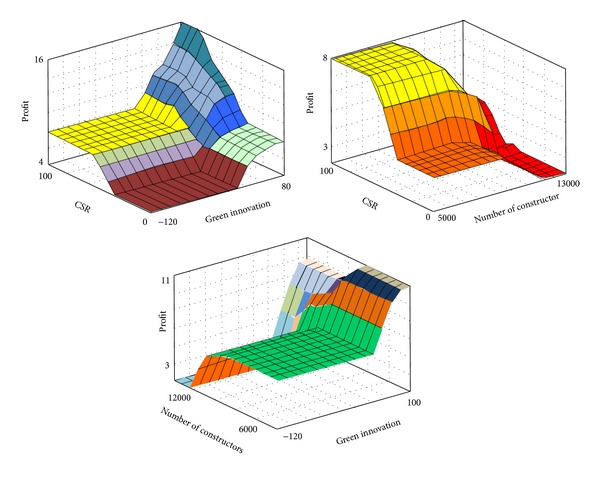
Inputs and output mapping.

The profit ratio was used to determine competitive advantage due to green innovation. The higher the quantitative value of the profit ratio, the better the competitive advantages of the contractor.  Figure 6 showed the 3D diagrams of input and output mapping.  Figure 6 may effectively help decision makers to make well-founded judgments based on an understanding of the association between the input and output in various scenarios.  Table 11 listed the optimal and worst quantitative output value, computed by the FLIS and the simulated cases. The input scenario in Table 11 was either the quantitative output value or the word used in natural language, such as good (high), ordinary (moderate), and poor (low). Therefore, according to the content in Table 11, the decision maker can assess and compare the advantages and disadvantages of different cases quantitatively and specifically decide the pros and cons of plans, such that rational decisions can be made systematically. 

## 5. Conclusions 

This study successfully combines four scientific methodologies to develop a contractor assessment model for facilitating green innovations. The system dynamics model predicts that the trend in the number of contractors in Taiwan will increase in the future. The increases will bring more competition to the construction industry and directly impact the contractor profits. Additionally, the construction industry will face more challenges, such as stricter factors in environmental protection, advances in technology, green transformation, and green innovation. These challenges will become important factors determining whether a contractor can survive in the market. The results of the three simulated cases (Case 1, Case 2, and Case 3) indicated that, in the future, contractors with better green innovation will be more profitable and more competitive in the market. The aforementioned lives of evidences suggest the importance of green innovation and the practical use of the proposed assessment model for the construction industry.

Every scientific methodology has its own basic hypothesis. This study complies with the fundamental hypothesis of each research methodology with appropriate applications of these hypotheses and methodologies. Various methodologies were employed in this study in a complementary manner, with each methodology addressing individual aspects of the problem. Therefore, this study explores the possibility of integrating various methodologies into industrial practices and demonstrates its applicability.

## Figures and Tables

**Table 1:** The regulations for the management of different classes of contractors.

Classification	Class C contractor	Class B contractor	Class A contractor
Capital	>3 million TWD	>15 million TWD	>100 million TWD
Establishment requirement		Registered as class C for two years and employed specialized engineering staff over last 5 years. Final contract amount over 100 million	Registered as class B for two years and employed specialized engineering staff over last 5 years. Final contract amount over 200 million TWD
Contract amount	<22.5 million TWD	<75 million TWD	no cap

**Table 2:** The meaning of variables used in the contractor rating system.

Variables	Meaning
A numbers	Number of class A contractors
B numbers	Number of class B contractors
C numbers	Number of class C contractors
Upgrading *N* of B	Number of class B upgrading to class A, annually
Upgrading *N* of C	Number of class C upgrading to class B, annually
Degrading *N* of A	Number of class A degrading to class B, annually
Degrading *N* of B	Number of class B degrading to class C, annually
Capi. develop. Time 1	Time needed for class C to upgrade to class B, in terms of capital development
Capi. develop. Time 2	Time needed for class B to upgrade to class A, in terms of capital development
B threshold	Contract amount required for class C to upgrade to class B
A threshold	Contract amount required for class B to upgrade to class A
A market share power	Market share power of class A contractors
B market share power	Market share power of class B contractors
C market share power	Market share power of class C contractors
Ave. employees of A	Average number of employees of class A contractors
Ave. employees of B	Average number of employees of class B contractors
Ave. employees of C	Average number of employees of class C contractors
Capital amount level of A	Average capital of class A contractors
Capital amount level of B	Average capital of class B contractors
Capital amount level of C	Average capital of class C contractors
Ave. revenue of A	Average revenue of class A contractors, per year
Ave. revenue of B	Average revenue of class B contractors, per year
Ave. revenue of C	Average revenue of class C contractors, per year
A market size	Market size, above 75 million
B market size	Market size, between 25~75 million
C market size	Market size, under 25 million

**Table 3:** Comparison between simulated and actual numbers of contractors.

Years	Simulated number of contractors	Actual number of contractors
1991	2,899	2,899
1992	3,335	3,236
1993	3,880	3,724
1994	4,660	4,490
1995	5,331	5,233
1996	6,281	6,158
1997	7,256	7,178
1998	8,457	8,464
1999	9,306	9,360
2000	10,528	10,606
2001	10,903	10,941
2002	13,139	13,254
2003	12,211	12,638
2004	8,900	8,822
2005	8,991	8,979
2006	9,146	9,089
2007	9,098	9,193
2008	9,056	9,198
2009	8,967	9,280
2010	8,922	9,454
2011	9,161	NA
2012	9,197	NA
2013	9,275	NA
2014	9,395	NA
2015	9,475	NA
2016	9,569	NA
2017	9,669	NA
2018	9,763	NA
2019	9,861	NA
2020	9,960	NA
2021	10,058	NA

**Table 4:** Weighting value of main criteria.

Comparison of construction/management, design, and procurement			
Attributes	Construction/Management	Design	Procurement
Construction/management	1	1	1 3/5
Design	1	1	1
Procurement	5/8	1	1
Eigenvector	0.39	0.33	0.28

**Table 5:** Weighting value of construction/management criteria.

Comparison of green technology, disposal of waste building materials, and green construction management			
Attributes	Green technology	Disposal of waste building materials	Green construction management
Green technology	1	4 1/2	1
Disposal of waste building materials	2/9	1	1
Green construction management	1	1	1
Eigenvector	0.49	0.20	0.31

**Table 6:** Weighting value of design criteria.

Comparison of reduction in energy consumption, green building and multidesign			
Attributes	Reduction in energy consumption	Green building	Multidesign
Reduction in energy consumption	1	1	2
Green building	1	1	2
Multidesign	1/2	1/2	1
Eigenvector	0.40	0.40	0.20

**Table 7:** Weighting value of procurement criteria.

Comparison of green procurement, green supply chain, green specifications			
Attributes	Green procurement	Green supply chain	Green specifications
Green procurement	1	1/3	1/3
Green supply chain	3	1	2
Green specifications	3	1/2	1
Eigenvector	0.14	0.52	0.34

**Table 8:** Weighting value of each criterion.

Main criteria (w_i_)	Subcriteria (w_i_ )	w_i_	W_i_%
Design (0.33)	Multidesign (0.2)	0.066	6.60%
	Reduction in energy consumption (0.4)	0.132	13.20%
	Green building (0.4)	0.132	13.20%
Procurement (0.28)	Green procurement (0.14)	0.0392	3.92%
	Green specifications (0.34)	0.0952	9.52%
	Green supply chain (0.52)	0.1456	14.56%
Construction/management (0.39)	Green construction management (0.31)	0.1209	12.09%
	Green technology (0.49)	0.1911	19.11%
	Disposal of waste building materials (0.20)	0.078	7.80%
*W*_*i*_ = *w*_*i*_∗100%		1	100%

**Table 9:** Most preferred point, constants, UF, and expected utility value for criteria.

Criterion *W*_*i*_%	*y*_*u*_	*y*_*L*_	*y*_*mi*_	*y*_*ma*_	*A*	*B*	Utility function *u*_*i*_(*y*_*i*_) = *Ay*_*i*_ + *B*	*u*_*ri*_∗(*W*_*i*_)	
								Worst	Optimal
Multidesign (6.60)	100	0	60	100	0.025	−1.50	*u*_*i*_(*y*_*i*_) = 0.025*y*_*i*_ − 1.5	−9.90	6.6
Reduction in energy consumption (13.20)	50	0	20	50	0.033	−0.66	*u*_*i*_(*y*_*i*_) = 0.033*y*_*i*_ − 0.66	−8.71	13.07
Green building (13.20)	100	0	60	100	0.025	−1.50	*u*_*i*_(*y*_*i*_) = 0.025*y*_*i*_ − 1.5	−19.80	13.20
Green procurement (3.92)	100	0	60	100	0.025	−1.50	*u*_*i*_(*y*_*i*_) = 0.025*y*_*i*_ − 1.5	−5.88	3.92
Green specifications (9.52)	100	0	60	100	0.025	−1.50	*u*_*i*_(*y*_*i*_) = 0.025*y*_*i*_ − 1.5	−14.28	9.52
Green supply chain (14.52)	100	0	50	100	0.020	−1	*u*_*i*_(*y*_*i*_) = 0.020*y*_*i*_ − 1.0	−14.52	14.52
Green construction management (12.09)	100	0	60	100	0.025	−1.50	*u*_*i*_(*y*_*i*_) = 0.025*y*_*i*_ − 1.5	−18.14	12.09
Green technology (19.11)	100	0	50	100	0.020	−1	*u*_*i*_(*y*_*i*_) = 0.020*y*_*i*_ − 1.0	−19.11	19.11
Disposal of waste building materials (7.8)	100	0	70	100	0.033	−4.00	*u*_*i*_(*y*_*i*_) = 0.033*y*_*i*_ − 2.33	−18.17	7.8
Expected utility value								−128.51	99.83

**Table 10:** Fuzzy set, fuzzy scale and output value.

Input scenario			Fuzzy output value	
Criteria	Value range	Fuzzy sets	Description	Fuzzy sets
*f*(*x*_1_) The number of constructor	6000 9000 13500	good ordinary poor	Quantitative value	Very good (16%↑) Good (12%↑) Ordinary (8%) Poor (6%↓) Very poor (4%↓) (0–20%)
*f*(*x*_2_) Green innovation	85 70 50 30 0 (−128~99)	Very good Good Ordinary Poor Very poor		
*f*(*x*_3_) Corporate social responsibility	100 60 0	Good Ordinary Poor		

**Table 11:** Optimal, worst output value, and simulated case.

Criteria	Optimal	Worst	Simulated case		
			Case 1	Case 2	Case 3
Number of constructors	Good	Poor	10000	10000	10000
Green innovation	Very good	Very poor	30	70 (good)	85 (very good)
Corprate social responsibility	Good	Poor	50	80	80
Output value (profit)	16.8	2.11	6.28	11.7	14.5
